# Statistical inference for extended or shortened phase II studies based on Simon’s two-stage designs

**DOI:** 10.1186/s12874-015-0039-5

**Published:** 2015-06-07

**Authors:** Junjun Zhao, Menggang Yu, Xi-Ping Feng

**Affiliations:** Department of General Dentistry, Shanghai Ninth People’s Hospital, College of Stomatology, Shanghai Jiao Tong University School of Medicine, 639 Zhi Zao Ju Road, Shanghai, 200011 P.R. China; Department of Biostatistics & Medical Informatics, University of Wisconsin, K6/446 CSC 600 Highland Ave., Madison, Wisconsin USA; Shanghai Ninth People’s Hospital, College of Stomatology, Shanghai Jiao Tong University School of Medicine, Shanghai, 639 Zhi Zao Ju Road200011 P.R. China

**Keywords:** Clinical trials, Simon’s two-stage designs, Likelihood, Phase II studies

## Abstract

**Background:**

Simon’s two-stage designs are popular choices for conducting phase II clinical trials, especially in the oncology trials to reduce the number of patients placed on ineffective experimental therapies. Recently Koyama and Chen (2008) discussed how to conduct proper inference for such studies because they found that inference procedures used with Simon’s designs almost always ignore the actual sampling plan used. In particular, they proposed an inference method for studies when the actual second stage sample sizes differ from planned ones.

**Methods:**

We consider an alternative inference method based on likelihood ratio. In particular, we order permissible sample paths under Simon’s two-stage designs using their corresponding conditional likelihood. In this way, we can calculate p-values using the common definition: the probability of obtaining a test statistic value at least as extreme as that observed under the null hypothesis.

**Results:**

In addition to providing inference for a couple of scenarios where Koyama and Chen’s method can be difficult to apply, the resulting estimate based on our method appears to have certain advantage in terms of inference properties in many numerical simulations. It generally led to smaller biases and narrower confidence intervals while maintaining similar coverages. We also illustrated the two methods in a real data setting.

**Conclusions:**

Inference procedures used with Simon’s designs almost always ignore the actual sampling plan. Reported P-values, point estimates and confidence intervals for the response rate are not usually adjusted for the design’s adaptiveness. Proper statistical inference procedures should be used.

## Background

Simon’s two-stage designs [1] are commonly used in phase II clinical trials, especially in cancer clinical trials. In a study with a Simon’s design, the null hypothesis is concerned with a response rate, *H*_0_:*π*≤*π*_0_. The power is calculated at some *π*_1_>*π*_0_. A Simon’s design is usually indexed by four numbers that represent the stage 1 sample size (*n*_1_), stage 1 critical value (*r*_1_), final sample size (*n*_*t*_) and final critical value (*r*_*t*_). In stage 1, a sample of size *n*_1_ is taken. If the number of successes *X*_1_ in stage 1 satisfies *X*_1_≤*r*_1_, the trial is stopped for futility; otherwise, an additional sample of size *n*_2_=*n*_*t*_−*n*_1_ is taken. Let *X*_2_ be the number of successes in stage 2, and let *X*_*t*_=*X*_1_+*X*_2_. If *X*_*t*_≤*r*_*t*_, futility is concluded; otherwise efficacy is concluded by rejecting *H*_0_. Softwares are available for calculating Simon’s two-stage designs, for example, from a website at the National Cancer Institute: http://linus.nci.nih.gov/brb/samplesize/otsd.html, from a website at the Department of Biostatistics of the Vanderbilt University: http://biostat.mc.vanderbilt.edu/wiki/Main/TwoStageInference, and from the NCSS/PASS package: http://www.ncss.com/.

Koyama and Chen [2] (hereafter KC) pointed out that the inference procedures used with Simon’s designs almost always ignore the actual sampling plan. Reported P-values, point estimates and confidence intervals for the response rate are not usually adjusted for the design’s adaptiveness. They outlined proper statistical inference procedures for studies based on the Simon’s two-stage designs.

Because the actual sample size of stage 2 may frequently differ from the planned one due to various reasons, KC also proposed a way to conduct a hypothesis testing when the stage 2 sample size is changed in a Simon’s design. They focused on the case of non-informative sample size change at the second stage. In other words, the actual stage 1 sample size always equals to the planned stage 1 sample size but the actual stage 2 sample size can differ from the planned stage 2 sample size. In addition, the decision to use a different sample size must be independent of the observed outcome data. Inference then needs to be made based on the actual data. This is in contrast to adaptive designs that can alter the sample size based on interim results. We restrict our attention to the same setting as KC although we believe our method can be extended.

The scenarios of non-informative sample size change or protocol deviation can arise quite frequently in practice. Shortening of stage 2 can occur in cases of early termination of study due to lack of funding, slow accrual, non-informative drop-outs, accrual of ineligible subjects, etc. Such shortening of stage 2 sample size can be reasonably assumed to be independent of the outcomes of the study. Extension of stage 2 can occur in cases of sites coordination error, over compensation for unevaluable or dropout patients, or administrative reasons.

In applying KC’s method, we found some difficulties in calculation for certain scenarios due to the discrete nature of the binomial distribution. In particular, in the case when the number of responders *x*_1_ at the first stage exceeds the final boundary *r*_*t*_ with an (unexpectedly) efficacious treatment. Because Simon’s two-stage design does not stop for early efficacy [1], the study would continue to the second stage. In this case, KC’s method breaks down. Another possible problem is for the case when we have no responders at the second stage, that is, *x*_2_=0. We give our detailed explanation after we review their method in the next section. We therefore introduce a different method for inference based on conditional likelihood. Besides the ability to make proper inference for the settings when KC’s method may be difficult to apply, our method is also seen to improve on statistical properties for many settings we have investigated.

Porcher and Desseaux [3] considered different approaches for point and confidence intervals estimation, as well as computation of p-values for the same setting as KC. In their methods, the rankings used for computing p-values were based on estimators instead of likelihood. They recommended the uniformly minimum variance unbiased estimator (UMVUE) as it exhibited good properties. In particular, when the second stage sample size is unaltered, they pointed out that the method based on UMVUE is equivalent to KC [3]. For this reason, our method should also improve on their methods.

In addition to [2, 3], other related works exist. Green and Dahlberg [4] were among the first who considered settings that accommodate a modified sample size in both stages even though the proposed analysis method was ad hoc. Masaki et al. [5] considered designs for a range of possible stage I and total sample size deviations from planned study. Li et al. [6] formulated a Bayesian approach with a modified sample size. Their method can have desirable frequentist properties under certain types of priors. Recently, Zeng et al. [7] considered computation improvement and proposed a normal approximation that is accurate even under small sample sizes.

## Methods

### Review of Koyama and Chen (2008)

The KC method centers mainly on the calculation of p-values. Throughout, use *P*_*π*_(*E*) to represent the probability of the event *E* at a specific *π*. Denote *x*_1_ and *x*_2_ as the actual observed numbers of responders at stage 1 and 2 of a study based on Simon’s two-stage design.

If *x*_1_≤*r*_1_, the trial is stopped early at the first stage due to futility. In this case, the p-value is given by $P_{\pi _{0}}[X_{1} \ge x_{1}|n_{1}]$, which can be easily computed from the binomial distribution with size *n*_1_ and success probability *π*_0_.

If *x*_1_>*r*_1_, the trial continues to the second stage. In this case, the p-value calculation is based on observed sample paths, given by

(1) $$ \sum_{x=r_{1}+1}^{n_{1}}P_{\pi_{0}}[X_{1}=x|n_{1}]P_{\pi_{0}}[X_{2}\geq x_{1}+x_{2}-x|n_{2}],  $$

where $P_{\pi _{0}}[X_{2}\geq x_{1}+x_{2}-x|n_{2}]$ represent more ‘extreme’ sample paths than the observed one given that *x*>*r*_1_ responses are observed at stage 1. The actual type I error and power are evaluated through

$$\begin{array}{@{}rcl@{}} P_{\pi}[\text{Reject}\ H_{0}] \,=\,\sum_{x = r_{1}+1}^{n_{1}} \!P_{\pi}[X_{1}\,=\,x|n_{1}] P_{\pi}[X_{2} > r_{t}-x \,|\, n_{2}] \end{array} $$

under *H*_0_ and *H*_1_, respectively. Let *A*(*x*,*n*_2_,*π*)≡*P*_*π*_[*X*_2_>*r*_*t*_−*x* | *n*_2_] be the conditional rejection rate of *H*_0_ at the end of stage 2 given *X*_1_=*x*. Then, the rejection rule at the end of stage 2, *x*_1_+*x*_2_>*r*_*t*_, is equivalent to

$$P_{\pi_{0}}[X_{2}\geq x_{2}|n_{2}] \le A(x_{1},n_{2},\pi_{0}), $$

where *A*(*x*_1_,*n*_2_,*π*_0_) serves as a conditional critical value.

When the actual sample size of stage 2, denoted by *n*^∗^, deviates from *n*_2_, *A*(*x*_1_,*n*_2_,*π*) can still be used as a conditional criterion for decision making. That is to reject *H*_0_ when

$$P_{\pi_{0}}[X_{2}\geq x_{2}|n_{2}^{*}] \le A(x_{1},n_{2},\pi_{0}). $$

However, with the presence of the second stage sample size deviation, the p-value cannot be directly extended from (1) because the observed total number of responses *x*_1_+*x*_2_ is not a good ranking determinant of ‘extremeness’ any more. In particular, KC gave a concrete example in which two different sample paths (*x*_1_,*x*_2_) and $(x_{1}^{*}, x_{2}^{*})$ with the same total number of responses ($x_{1}^{*}+x_{2}^{*}=x_{1}+x_{2}$) and the same deviated sample size $n_{2}^{*}$ of stage 2 may lead to different conclusions about the hypothesis. Therefore, Koyama and Chen [2] proposed the following way of calculating p-value.

- Find *π*^∗^ such that $A(x_{1},n_{2},\pi ^{*})=P_{\pi _{0}}[X_{2}\geq x_{2}|n_{2}^{*}]$.
- Compute the p-value by
  $$\begin{array}{@{}rcl@{}} \sum_{x = r_{1}+1}^{n_{1}} P_{\pi_{0}}[X_{1}=x|n_{1}]A(x,n_{2},\pi^{*}). \end{array} $$

One difficulty with this way of calculation is when *x*_1_>*r*_*t*_. Although infrequent, this happens when the investigational treatment is unexpectedly efficacious. Because Simon’s two-stage designs do not stop for early efficacy [1], the study continues to the second stage. In this case, we have *A*(*x*_1_,*n*_2_,*π*)≡1 for any *π*. Therefore *π*^∗^ can not be determined from step (a) above and the algorithm breaks down.

Another possible problem is for the case when we have *x*_2_=0. In this case, $P_{\pi _{0}}[X_{2}\geq x_{2}|n_{2}^{*}] \equiv 1$ for any $n_{2}^{*}$. When *x*_1_≤*r*_*t*_, this corresponds to the solution *π*^∗^=1. Therefore the corresponding p-value is independent of $n_{2}^{*}$ and equals to $\sum _{x = r_{1}+1}^{n_{1}} P_{\pi _{0}}[X_{1}=x] = P_{\pi _{0}}[X_{1}> r_{1}]$. This may not be sensible as it is independent of both observed number of response *x*_1_ and of the actual second stage sample size $n_{2}^{*}$. We therefore introduce a different method for inference based on likelihood.

### Likelihood based construction of confidence intervals

We extend the existing likelihood based inference for two-stage and multiple stage trials [8–12] to our setting for construction of p-values and confidence intervals. In particular, we order permissible sample paths under Simon’s two-stage designs using their corresponding conditional likelihood. In this way, we can calculate p-values using the common definition: the probability of obtaining a test statistic value at least as extreme as that observed under *H*_0_.

Let *M* denote the stopping stage, and let *S*_*M*_ denote the total number of responders accumulated up to the stopping stage. That is, *S*_*M*_=*X*_1_ when *M*=1 and *S*_*M*_=*X*_1_+*X*_2_ when *M*=2. Similarly, let *N*_*M*_ be total sample size of the study. The probability mass function of the random vector (*M*;*S*_*M*_) is given by

(2) $$\begin{array}{@{}rcl@{}} f(m, s_{m} |\pi)\,=\, \left\{ \begin{array}{ll} {n_{1}\choose s_{m}} \pi^{s_{m}}(1 - \pi)^{n_{1}-s_{m}} & \quad m=1 \\ \sum_{x_{1}=(r_{1}+1)\vee (s_{m}-n_{2})}^{s_{m} \wedge n_{1}} {n_{1}\choose x_{1}} {n_{2}\choose s_{m}-x_{1}} \pi^{s_{m}}(1 - \pi)^{n_{1}+n_{2}-s_{m}} & \quad m=2 \end{array} \right. \end{array} $$

where ∧ takes the minimum and ∨ takes the maximum of its arguments. Jung and Kim [8] showed that (*M*,*S*_*M*_) is complete and sufficient for *π*. The MLE of *π* is therefore $\hat {\pi }=S_{M}/N_{M}$. However the MLE is biased [11, 13]. Based on the fact that *X*_1_/*n*_1_ is always unbiased estimator for the true probability *π*, Jung and Kim [8] derived the UMVUE of *π* to be

(3) $$\begin{array}{@{}rcl@{}} \tilde{\pi}= \left\{ \begin{array}{ll} \frac{x_{1}}{n_{1}} & m=1\\ \\ \frac{\sum_{x_{1}=(r_{1}+1)\vee (s_{m}-n_{2})}^{s_{m}\wedge n_{1}}{n_{1}-1 \choose x_{1}-1}{n_{2} \choose s_{m}-x_{1}}}{\sum_{x_{1}=(r_{1}+1)\vee (s_{m}-n_{2})}^{s_{m}\wedge n_{1}}{n_{1} \choose x_{1}}{n_{2} \choose s_{m}-x_{1}}}& m=2 \end{array} \right. \end{array} $$

The existence of the UMVUE $\tilde {\pi }$ also facilitates the determination of confidence intervals. In particular, an exact (1−*α*)*%* confidence interval (*π*_*L*_,*π*_*U*_) for *π* is given by

$$\begin{array}{@{}rcl@{}} Pr(\tilde{\pi}(M, S_{M}) \ge \tilde{\pi}(m,s_{m}) |\pi=\pi_{L}) = \alpha/2 \end{array} $$

and

$$\begin{array}{@{}rcl@{}} Pr(\tilde{\pi}(M, S_{M}) \ge \tilde{\pi}(m,s_{m}) |\pi=\pi_{U}) = 1-\alpha/2. \end{array} $$

Jung and Kim [8] showed that such ordering of the sample space by the UMVUE is the same as that by Jennison and Turnbull [14]. Chang and O’Brien [12] showed that likelihood ratio based construction is more efficient and led to smaller average CI length.

When there is study extension or shortening, the second stage sample size *n*_2_ becomes a random variable. The likelihood can depend on the probability that *n*_2_ obtains a specific value $n_{2}^{*}$. However, in the case when such change of sample size is not related to *π*, the above likelihood can be viewed as the conditional likelihood given the observed value of $n_{2}^{*}$ and therefore can be used to make inference. The UMVUE takes the same format as in (3) except with $n_{2}^{*}$ in place of *n*_2_.

The likelihood ratio test of *H*_0_:*π*=*π*_0_ vs. *H*_1_:*π*≠*π*_0_ is based on

(4) $$\begin{array}{@{}rcl@{}} T(M, S_{M}, \pi_{0}) = \frac{\hat{\pi}^{S_{M}}(1-\hat{\pi})^{N_{M}-S_{M}}}{\pi_{0}^{S_{M}}(1-\pi_{0})^{N_{M}-S_{M}}}, \end{array} $$

where $\hat {\pi }=S_{M}/N_{M}$. Under *H*_0_, any path (*m*,*s*_*m*_) that has larger likelihood ratio is considered to be more ‘extreme’ against *H*_0_. Therefore, the probability of observing (*M*,*S*_*M*_) or more extreme paths is

$$\sum_{\{(m,s_{m}): T(m, s_{m}, \pi_{0}) > T(M, S_{M}, \pi_{0})\}} f(m,s_{m}|\pi_{0}). $$

After correcting for the discreteness of the binomial distribution by a fraction of the probability of (*M*,*S*_*M*_), the p-value is proposed to be

(5) $$\begin{array}{@{}rcl@{}}{\fontsize{8.3pt}{12pt}\selectfont{ \begin{aligned} {}P_{\pi_{0}} \equiv \sum_{\{(m,s_{m}): T(m, s_{m}, \pi_{0}) > T(M, S_{M}, \pi_{0})\}} f(m,s_{m}|\pi_{0}) + 0.5f(M, S_{M}|\pi_{0}). \end{aligned}}} \end{array} $$

The acceptance region defined as $\{\pi _{0}: P_{\pi _{0}} \ge \alpha \}\phantom {\dot {i}\!}$ can be used to form the limits of a (1−*α*)*%* confidence interval of *π*. Note that it is possible that such a defined region may not be an interval. However, such case is rare and has minimal impact on the confidence interval performance [12].

## Results and discussion

### Simulation study

**Fig. 1:**
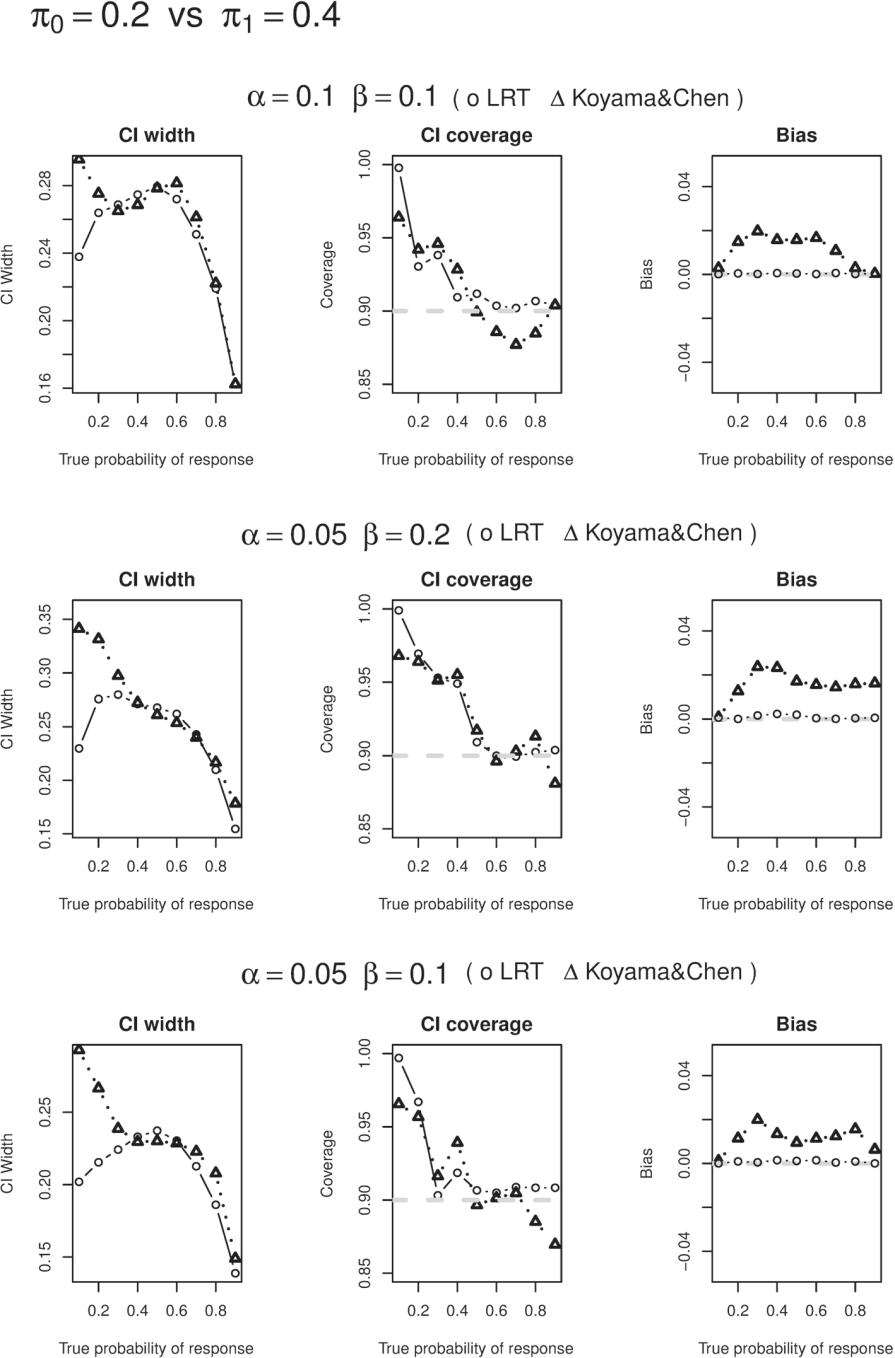
Confidence interval width comparison is based on studies made to the second stage; Coverage is to be compared with 90 %; Bias is the absolute value of difference between the estimate and true probability of response

**Fig. 2:**
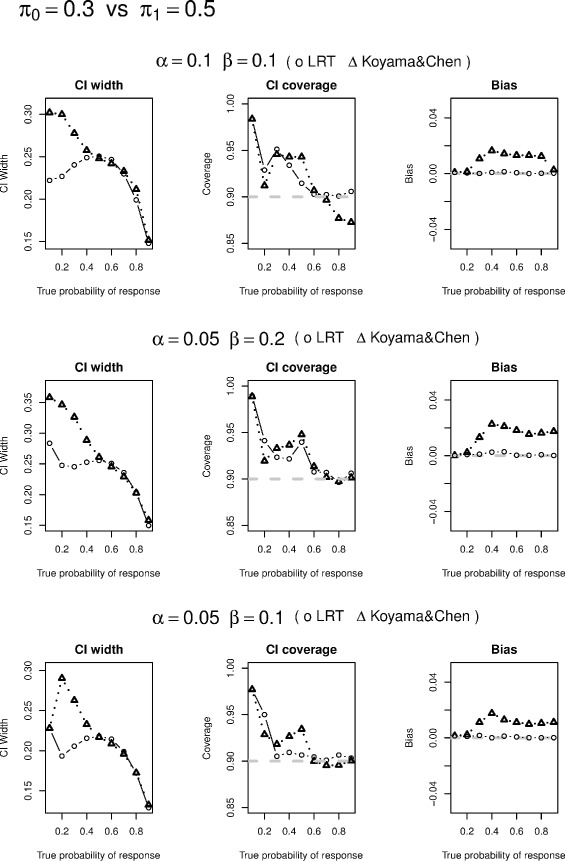
Confidence interval width comparison is based on studies made to the second stage; Coverage is to be compared with 90 %; Bias is the absolute value of difference between the estimate and true probability of response

**Fig. 3:**
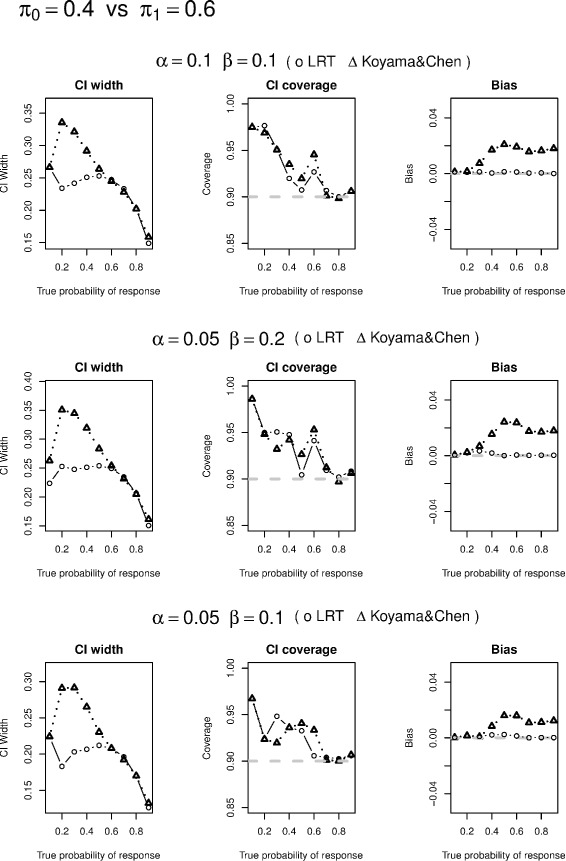
Confidence interval width comparison is based on studies made to the second stage; Coverage is to be compared with 90 %; Bias is the absolute value of difference between the estimate and true probability of response

**Fig. 4:**
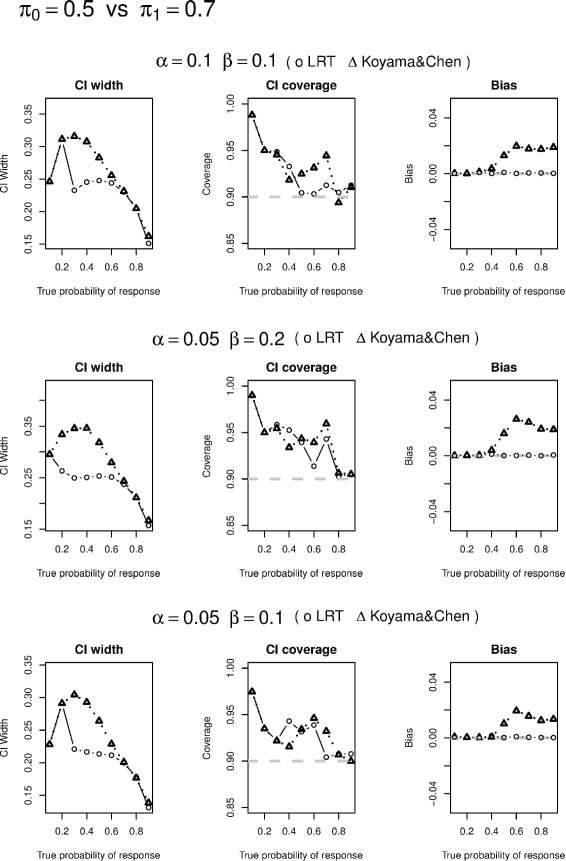
Confidence interval width comparison is based on studies made to the second stage; Coverage is to be compared with 90 %; Bias is the absolute value of difference between the estimate and true probability of response

We conduct simulation studies to evaluate likelihood ratio test based CI construction, conditional likelihood based UMVUE, and compare their performances with approaches of Koyama and Chen [2]. In particular, we selected the designs from Tables one and two in Simon’s paper [1] and simulated 5,000 data sets based on various values of *π*. If a simulated study continues to the 2nd stage under the specified design, the actual sample size at the second stage of the study $n_{2}^{*}$ is generated via an equal-probability multi-nomial distribution that range from *n*_2_/3 to 1.5*n*_2_. We have also examined other possible ranges of $n_{2}^{*}$ and found similar results. We only report 90 % CI widths and coverage as well as the actual power from the two methods in Tables 1, 2 and 3 and visualized the comparison of the corresponding CI widths, CI coverage, and bias in Figs. 1, 2, 3 and 4. Since the two methods yield same CIs in the first stage, we only present the CI width comparison for studies that are made to the 2nd stage in our simulation. From the tables, we see that the average CI width based on conditional likelihood are either similar to or smaller than those based on Koyama and Chen [2] in most cases. In some cases, the improvement can be quite significant (Figs. 1, 2, 3 and 4).

**Table 1:** Ninety percent CI width and actual power based on studies made to the 2nd stage (*α*=0.05, *β*=0.1)

	Width		Coverage		Actual power	
*π*_*true*_	LR	KC	LR	KC	LR	KC
Design 1 (0.2 vs 0.4)						
(*r*_1_,*n*_1_,*r*2,*n*)=(3,17,10,37)						
0.1	.257	.260	99.7	96.6	0.3	0.0
0.2	.271	.289	94.5	93.0	3.1	4.7
0.3	.250	.260	90.1	92.7	38.4	44.3
0.4	.238	.235	91.2	94.3	85.7	86.7
0.5	.236	.230	89.9	88.6	98.5	98.6
0.6	.229	.228	90.2	89.0	100.0	100.0
0.7	.211	.222	88.8	88.2	100.0	100.0
0.8	.184	.208	90.6	89.2	100.0	100.0
Design 2 (0.3 vs 0.5)						
(*r*_1_,*n*_1_,*r*_2_,*n*)=(7,22,17,46)						
0.1	.227	.227	97.5	97.5	0.0	0.0
0.2	.285	.289	95.1	92.7	0.1	0.1
0.3	.283	.301	90.0	91.5	2.1	4.5
0.4	.253	.265	87.9	91.1	33.3	43.3
0.5	.225	.224	90.0	92.6	79.6	85.1
0.6	.214	.208	89.7	88.9	98.4	98.6
0.7	.198	.195	92.4	91.6	99.8	99.8
0.8	.172	.172	90.8	90.3	100.0	100.0
Design 3 (0.4 vs 0.6)						
(*r*_1_,*n*_1_,*r*_2_,*n*)=(7,18,22,46)						
0.1	.219	.219	97.2	97.2	0.0	0.0
0.2	.286	.286	93.0	93.0	0.2	0.0
0.3	.315	.319	95.9	92.5	0.4	0.1
0.4	.300	.317	93.4	93.4	1.7	3.7
0.5	.258	.270	93.3	94.2	31.2	42.0
0.6	.218	.218	91.7	94.2	82.5	87.2
0.7	.198	.194	92.2	91.0	98.8	98.8
0.8	.170	.170	90.2	90.1	100.0	100.0
Design 4 (0.5 vs 0.7)						
(*r*_1_,*n*_1_,*r*_2_,*n*)=(11,21,26,45)						
0.1	.231	.231	95.9	95.9	0.0	0.0
0.2	.292	.292	92.6	92.6	0.0	0.0
0.3	.327	.327	94.1	93.8	0.0	0.0
0.4	.342	.346	94.6	92.0	0.1	0.0
0.5	.316	.331	93.5	93.7	1.5	4.4
0.6	.259	.271	91.9	92.1	26.8	37.5
0.7	.209	.210	89.3	92.9	80.5	85.7
0.8	.177	.176	88.3	88.5	98.6	99.2

**Table 2:** Ninety percent CI width and actual power based on studies made to the 2nd stage (*α*=0.1, *β*=0.1)

	Width		Coverage		Actual power	
*π*_*true*_	LR	KC	LR	KC	LR	KC
Design 1 (0.2 vs 0.4)						
(*r*_1_,*n*_1_,*r*2,*n*)=(3,13,12,43)						
0.1	.265	.270	99.7	96.6	0.1	0.0
0.2	.287	.292	93.2	94.5	4.6	8.4
0.3	.278	.275	94.2	94.6	40.4	47.8
0.4	.276	.270	91.3	93.4	81.3	86.2
0.5	.278	.277	89.3	87.8	97.3	97.5
0.6	.270	.281	91.1	89.4	99.9	99.8
0.7	.250	.260	90.2	87.7	100.0	100.0
0.8	.218	.221	91.4	88.6	100.0	100.0
Design 2 (0.3 vs 0.5)						
(*r*_1_,*n*_1_,*r*_2_,*n*)=(5,15,18,46)						
0.1	.239	.239	98.6	98.6	0.0	0.0
0.2	.298	.302	92.2	90.5	0.2	0.2
0.3	.299	.311	94.4	93.6	2.9	6.4
0.4	.273	.280	91.8	92.9	34.6	50.1
0.5	.256	.254	90.1	93.2	79.1	87.0
0.6	.246	.241	87.9	88.2	98.0	99.1
0.7	.228	.232	90.1	88.9	100.0	100.0
0.8	.198	.211	91.5	89.6	100.0	100.0
Design 3 (0.4 vs 0.6)						
(*r*_1_,*n*_1_,*r*_2_,*n*)=(7,16,23,46)						
0.1	.265	.265	97.0	97.0	0.0	0.0
0.2	.337	.338	98.3	98.0	0.5	0.0
0.3	.354	.365	95.1	95.4	0.9	0.2
0.4	.328	.347	93.6	94.9	5.4	7.0
0.5	.289	.298	89.2	90.3	37.4	45.7
0.6	.258	.256	92.8	95.0	83.4	85.6
0.7	.235	.231	90.9	90.7	98.9	98.8
0.8	.206	.205	90.2	88.9	100.0	100.0
Design 4 (0.5 vs 0.7)						
(*r*_1_,*n*_1_,*r*_2_,*n*)=(8,15,26,43)						
0.1	.246	.246	99.1	99.1	0.0	0.0
0.2	.310	.310	95.7	95.7	0.0	0.0
0.3	.348	.349	93.6	93.4	0.0	0.0
0.4	.361	.366	93.1	91.4	0.5	0.4
0.5	.336	.349	91.3	93.3	5.7	8.3
0.6	.289	.297	89.9	92.7	37.4	43.9
0.7	.242	.243	89.6	93.5	85.2	87.1
0.8	.204	.203	89.1	90.0	99.0	99.4

**Table 3:** Ninety percent CI width and actual power based on studies made to the 2nd stage (*α*=0.05, *β*=0.2)

	Width		Coverage		Actual power	
*π*_*true*_	LR	KC	LR	KC	LR	KC
Design 1 (0.2 vs 0.4)						
(*r*_1_,*n*_1_,*r*2,*n*)=(4,19,15,54)						
0.1	.313	.316	99.7	97.5	0.0	0.0
0.2	.342	.358	95.8	94.9	0.0	4.1
0.3	.328	.338	95.4	95.1	1.1	36.3
0.4	.295	.297	94.3	94.4	11.3	74.1
0.5	.272	.265	91.5	91.8	52.4	94.8
0.6	.263	.254	91.2	90.2	89.7	99.3
0.7	.243	.240	89.4	88.5	99.7	100.0
0.8	.208	.217	90.4	92.0	100.0	100.0
Design 2 (0.3 vs 0.5)						
(*r*_1_,*n*_1_,*r*_2_,*n*)=(8,24,24,63)						
0.1	.291	.291	99.0	98.6	0.0	0.0
0.2	.356	.362	94.2	92.4	0.1	0.0
0.3	.352	.375	92.0	92.9	1.6	3.4
0.4	.318	.339	91.9	94.1	20.9	31.3
0.5	.279	.285	94.9	95.7	66.4	76.4
0.6	.256	.251	89.9	90.8	95.1	96.8
0.7	.235	.229	90.2	90.4	99.0	99.0
0.8	.205	.204	90.3	89.5	100.0	100.0
Design 3 (0.4 vs 0.6)						
(*r*_1_,*n*_1_,*r*_2_,*n*)=(11,25,32,66)						
0.1	.287	.287	98.1	98.1	0.0	0.0
0.2	.357	.357	95.2	94.9	0.0	0.0
0.3	.386	.394	94.7	92.5	0.4	0.1
0.4	.370	.390	94.7	94.2	3.2	4.0
0.5	.325	.342	89.9	92.5	24.7	29.9
0.6	.274	.278	91.7	93.1	73.4	76.0
0.7	.241	.238	89.6	90.0	94.6	94.8
0.8	.209	.207	90.2	88.4	100.0	100.0
Design 4 (0.5 vs 0.7)						
(*r*_1_,*n*_1_,*r*_2_,*n*)=(13,24,36,61)						
0.1	.297	.297	98.3	98.3	0.1	0.0
0.2	.365	.365	94.8	94.8	0.1	0.0
0.3	.408	.409	94.8	94.1	0.2	0.0
0.4	.419	.427	94.6	93.3	0.5	0.0
0.5	.388	.408	94.5	94.7	3.1	3.9
0.6	.334	.350	89.9	92.8	23.9	28.8
0.7	.265	.270	93.9	95.6	71.5	74.2
0.8	.214	.214	89.8	89.9	97.2	97.5

We also compare CI coverage and bias based on all simulation studies including those stopped after the first stage. We see that the CI coverage are similar between the two methods. The conditional likelihood UMVUE has uniformly smaller biases than the estimate based on Koyama and Chen [2], especially when the underlying true probability is large.

### Real example

Advanced hepatobiliary cancers have a poor prognosis, in part complicated by underlying liver dysfunction. Although surgical resection and liver transplantation can be curative for select patients, those with advanced disease have few treatment options with survival rates of 6-12 months. GI06-101 was a multi-institutional study conducted by the Hoosier Oncology Group aimed to assess the efficacy of erlotinib (Tarceva, OSI-774; OSI Pharmaceuticals, Melville, NY) in combination with docetaxel in refractory hepatobiliary cancers [15]. Due to similarly poor outcomes and few existent treatment options for refractory disease at the time of this study’s design in 2006, both hepatocellular cancers and biliary tract cancers were included.

The primary end point of this trial was the rate of progression free survival (PFS) at 16 weeks. PFS was defined as time from the start of treatment until disease progression or death of any cause, whichever occurred first. A Simon optimal two-stage design tested the hypothesis that the 16-week PFS is *π*_0_≤15 *%* (clinically inactive) versus the alternative of *π*_1_≥30 *%* (warranting further study). The design used 0.10 as the level of significance and 80 % as power. This led to *n*_1_=19, *r*_1_=3, *n*_*t*_=39, and *r*_*t*_=8.

Among the 19 patients of the first stage, 8 were progression free at 16-week. The study went on to the second stage and was terminated due to lack of funding after recruiting 6 patients. Among these 6 patients, 4 were progression free at 16-week. Therefore we have $n_{2}^{*}=6$, *x*_1_=8, and *x*_2_=4. The resulting estimate for 16-week PFS rate is 0.435 with 90 % confidence interval (0.271,0.605) based on Koyama and Chen’s method, compared with 0.48 with 90 % confidence interval (0.322,0.646) based on the conditional likelihood method. The conditional likelihood based estimate is larger and has shorter CI width.

## Conclusions

Koyama and Chen [2] considered statistical inference problem for phase II studies based on Simon’s two-stage designs when there are study deviations at the second stage. We propose an alternative method for such problem based on likelihood principle. In addition to provide inference for a couple of scenarios where Koyama and Chen’s method breaks down, the resulting estimate appears to have certain advantage in terms of bias magnitude and confidence interval width in many cases.

Sample size change can also happen in the first stage [4, 16]. Our method of inference should be applicable if such change is not related to the actual outcome. There is also recent research on adaptive Simon’s two-stage designs [17] where the second stage sample size is decided at the end of stage 1 based on observed responses. The decision can be to extend the study because there are fewer positive responses than expected or to shorten the study simply because there are more positive responses than expected. Our method should also be applicable. However the whole likelihood needs to be used that incorporates the mechanism of the second stage sample size determination.
